# P-1786. "The Cloacae Conundrum: Genomic and Phenotypic Trail of Novel Enterobacter cloacae "

**DOI:** 10.1093/ofid/ofaf695.1955

**Published:** 2026-01-11

**Authors:** Nizamuddin Ahmed Mohammed, Mamta Puraswani, Bharat Das, Vanlal Tluanpuii, Madhavi Kirti, Rajathadri Hosur Ravikumar, Sushma sagar, Parul Singh, Kamraan farooque, Purva Mathur

**Affiliations:** AIIMS New Delhi, Delhi, Delhi, India; AIIMS, New Delhi, New Delhi, Delhi, India; AIIMS, New Delhi, New Delhi, Delhi, India; AIIMS, New Delhi, New Delhi, Delhi, India; AIIMS, New Delhi, New Delhi, Delhi, India; AIIMS, New Delhi, New Delhi, Delhi, India; AIIMS, New Delhi, New Delhi, Delhi, India; AIIMS, New Delhi, New Delhi, Delhi, India; AIIMS, New Delhi, New Delhi, Delhi, India; AIIIMS, New Delhi, New Delhi, Delhi, India

## Abstract

**Background:**

This study examines colistin-resistant *Enterobacter cloacae* isolates, focusing on genomic and phenotypic resistance mechanisms, including chromosomal mutations and efflux pumps. It also addresses challenges in susceptibility testing and genotype-phenotype discordance

**Figure:**
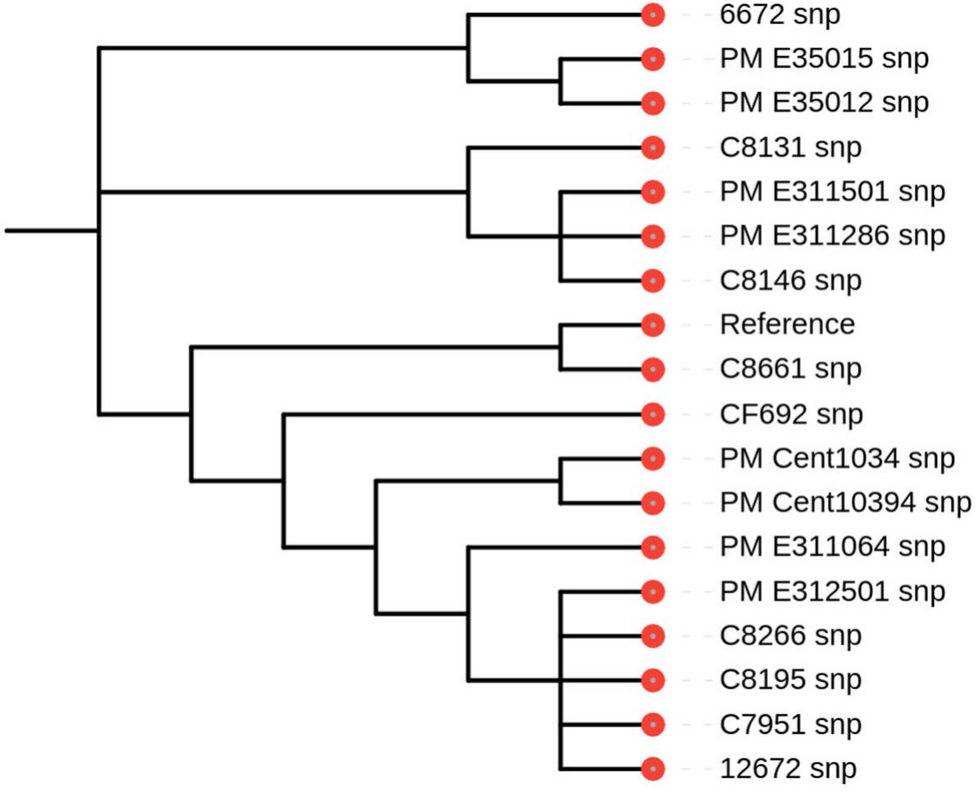
Phylogenetic tree (SNP based) of E. cloacae

**Methods:**

From Aug 2023 to Apr 2025, 235 *E.loacae* isolates were collected from various clinical samples; 56 showed colistin resistance by BMD. AST for 13 antibiotics was performed using disk diffusion. Colistin MICs were compared between VITEK 2 and BMD. WGS was performed on 18 resistant isolates using Illumina and Oxford Nanopore. Hybrid assemblies were generated via Unicycler and annotated using pubMLST, Abricate, VariantFinder, and PlasmidFinder databases

**Results:**

Among 235 *E. cloacae* isolates, 56 (23.8%) were colistin-resistant by BMD. Most patients were ICU-admitted, median age 30 years, with 66% male. These 56 isolates showed 100% resistance to amoxicillin-clavulanate and 69% to cefuroxime, yet remained fully susceptible to 13 other antibiotics. VITEK 2 consistently underestimated colistin MICs compared to BMD (e.g., 0.5 vs. 32 µg/mL). WGS of 18 resistant isolates revealed genome sizes of 4.7–5.0 Mb, all with novel sequence types, 103 AMR genes (no *mcr*), and universal *oqxA/B* and *blaACT*. Plasmids were detected in 72.2% (13/18), predominantly IncFIB(K)_1_Kpn3. Universal virulence genes *ompA* and *csgG* suggest enhanced pathogenicity.

**Conclusion:**

A study of 235 *E. cloacae* isolates found 56 (23.8%) were colistin-resistant by BMD. The median age was 30 years, with 66% male, and most isolates were from ICU patients. Antibiotic susceptibility testing showed 100% susceptibility to 13 antibiotics, including tigecycline and carbapenems, but 100% resistance to amoxicillin-clavulanate and 69% to cefuroxime. Colistin MICs showed discrepancies between VITEK 2 and BMD, with BMD reporting higher MICs (16– >32 µg/mL). WGS of 18 isolates revealed novel STs, 103 AMR genes, no mcr genes, and universal oqxA/B and blaACT. Plasmids (IncFIB(K)_1_Kpn3) were found in 72.2% of isolates, and virulence genes (ompA, csgG) were present in all, with *entA* and *entB* in 22.2%. These findings suggest genetically distinct, resistant strains,novel STs may drive heteroresistance and phenotypic-genotypic discordance.

**Disclosures:**

All Authors: No reported disclosures

